# Incidence and geographic distribution of retinoblastoma in Ethiopia

**DOI:** 10.1186/s12886-023-02980-8

**Published:** 2023-05-23

**Authors:** Sadik Taju Sherief, Gadisa Asfaw, Aemero Abateneh, Solomon Takewe, Diriba Fufa, Teshager Wondale, Temesgen Takele, Helen Dimaras

**Affiliations:** 1 Department of Ophthalmology, School of Medicine, College of Health Sciences, Addis Ababa University, P.O. Box 9086, Addis Ababa, Ethiopia; 2 Department of Ophthalmology, Bahir Dar University, Bahir Dar, Ethiopia; 3 Department of Pediatrics, Jimma University, Jimma, Ethiopia; 4 Department of Ophthalmology, University of Gondar, Gondar, Ethiopia; 5 Department of Ophthalmology, Hawassa University, Hawassa, Ethiopia; 6 Child Health Evaluative Sciences Program and Centre for Global Child Health, SickKids Research Institute, Toronto, Canada; 7 Department of Ophthalmology & Vision Sciences, The Hospital for Sick Children, University of Toronto, Toronto, Canada

**Keywords:** Retinoblastoma, Incidence, Birth cohort analysis, Ethiopia

## Abstract

**Introduction:**

Retinoblastoma is the most frequent intraocular malignancy of the eye in children, occurring in early childhood. Based on global estimates, Ethiopia is expected to observe over 200 new retinoblastoma cases per year, however without a cancer registry, this number is difficult to confirm. Therefore, the goal of the study was to determine the incidence and geographic distribution of retinoblastoma in Ethiopia.

**Methods:**

A retrospective medical chart review of clinically diagnosed new retinoblastoma patients between January 1, 2017 - December 31, 2020, in four public Ethiopian tertiary hospitals was performed. The incidence of retinoblastoma was calculated by a birth-cohort analysis.

**Results:**

There were 221 retinoblastoma patients observed in the study period. The incidence of retinoblastoma was found to be 1 in 52,156 live births. Incidence varied among different regions of Ethiopia.

**Conclusion:**

The incidence of retinoblastoma observed in this study is likely an underestimate. It is possible that patients were undercounted because they were seen outside of the 4 main retinoblastoma treatment facilities included in this facility, or they experienced barriers to accessing care. Our study suggests a need for a nationwide retinoblastoma registry and more retinoblastoma treatment centers in the country.

**Supplementary Information:**

The online version contains supplementary material available at 10.1186/s12886-023-02980-8.

## Introduction

Retinoblastoma (RB) is a rare tumor of childhood arising from retina and representing 3% of all childhood malignancies. It is the most frequent intraocular malignancy of the eye in childhood occurring in early childhood; two-thirds are diagnosed before 2 years of age, and 95% before 5 years [1]. Retinoblastoma is a cancer with a genetic etiology and arises from mutations in both alleles of the retinoblastoma gene (*RB1*). The disease may occur in a heritable (germline mutation) and non-heritable form (somatic mutation) [2, 3].

The annual incidence rate is 6 to 12 cases per 1 million children younger than 5 years in Europe, the United States, and Australia [4–6]. In Argentina, the incidence was calculated to be 5 cases per 1 million children under 14 years of age [7]. Any incidence estimate of RB based on the number of children of particular age, will be influenced by differences in child and adolescent mortality between countries, the more so the wider the age cohort considered [8]. A Northern European study from Sweden and Finland, which have comprehensive national cancer registries, explicitly shows that RB incidence rates based on age cohorts are the most volatile; the most stable ones cumulate children to their birth cohorts [9].

Based on birth cohort analysis, the incidence in various well-studied population groups around the world varies from 1 to 15,000 to 1 in 20,000 live births, with an estimated 8,000 children diagnosed each year worldwide [9–12]. The expected number of patients with RB annually per country is most accurately calculated by multiplying the global RB incidence (1 in 16,000–18,000 live births per year) by the forecast number of surviving infants (birth rate minus infant mortality rate [4, 8]. There is no validated evidence of geographical or racial variations of RB incidence.

As the epidemiology and geographic distribution of RB are largely unknown in Ethiopia, the precise risk factors are poorly understood. Anecdotal evidence suggests that RB patients may come from a certain cluster of regions in the country. We were interested to find out if the incidence of RB in these areas fluctuates, in which case this research will lay the groundwork for further studies. Thus, this study aimed to evaluate the incidence rates and geographic distribution of retinoblastoma in Ethiopia during the study period.

## Materials and methods

### Study Design and setting

The study is a retrospective cross-sectional multi-center chart review of clinically diagnosed new RB patients at Menelik II Hospital, Jimma University Hospital, University of Gondar Hospital and Hawassa University Hospitals between January 1, 2017 to December 31, 2020.

The centers provide comprehensive ophthalmology services including diagnosis, medical and surgical management of retinoblastoma. These are Ethiopia’s main RB treatment centres in the northwest, central, southern, and southwestern parts of the country. All Ethiopian RB patients are expected to be referred to and seek treatment at these facilities.

### Data Collection

New RB patients presenting during the study period were identified through review of the departmental records of all patients presenting to the pediatric ophthalmology clinics at the included study sites. The following information were extracted from each patient medical record: birthdate, diagnostic details, date of diagnosis, sex, address (which included region and zone), laterality, and family history of RB. Referral pathway was also consulted to help identify duplicates among the 4 study sites.

### Data Analysis

Data was coded, entered and analyzed using SPSS version 23.0 statistical software. The initial plan was to obtain data on population at risk, live birth of study period and neonatal death from Ethiopian Statistical Agency, an agency responsible for conducting a population and Housing Census (PHC) every 10 years. The last PHC was conducted in 2007 and it was not possible to get updated information. Hence, we relied on data from World Bank country profiles (https://data.worldbank.org/country/ethiopia) which include a yearly estimate of total population, birth rate, neonatal mortality rate. Using these data, the number of live births per given year was calculated using the following formula: # Of live births in Year X = (total population) *[(birth rate) *(1-infant mortality rate), as previously described [9, 13]. Frequency per zone, incidence per geographic areas were also calculated by population at risk.

## Results

### Study subjects

**Fig. 1:**
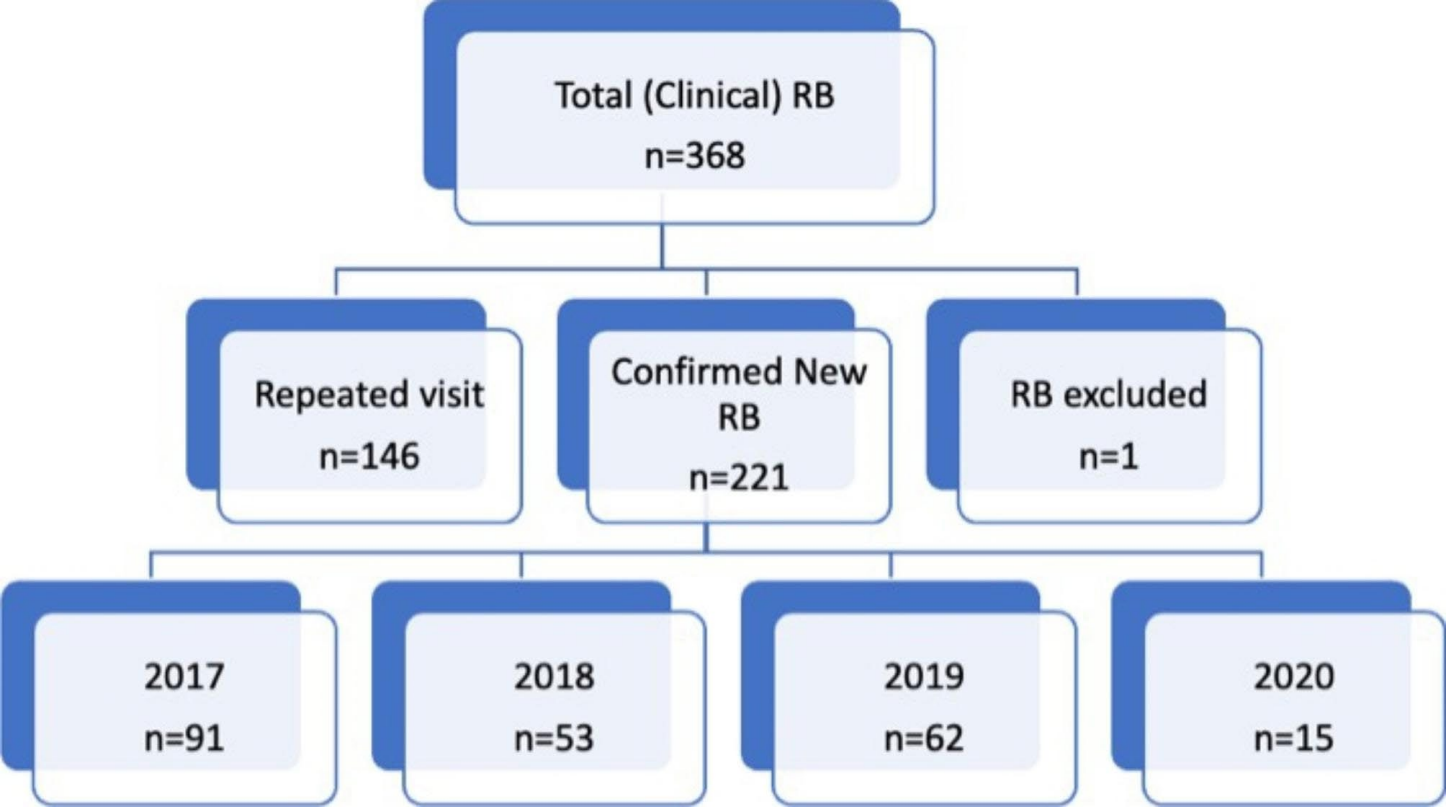
Number of Retinoblastoma patients in Ethiopia from 2017–2020

During the study period, 368 RB patient records were accessed at all four participating centres. Of these, 146 were duplicate cases, and 1 was excluded due to a misdiagnosis. A total of 221 unique subjects were included in the study (shown in Fig. 1).

The patient distribution among the centers were: 159 (71.9%) seen at Menelik II Hospital followed by 32 (14.5%) from Jimma University Hospital and 20 (9.0%) from University of Gondar Hospital and 10 (4.5%) from Hawassa University Hospital (shown in Table 1).

### Study subject demographics

One hundred and fifteen (52%) study subjects were male, and 106 (48%) were female, resulting in a male: female ratio of 1.08:1. Only 11 (5%) study subjects had a positive family history of retinoblastoma and 83.7% presented with unilateral disease (shown in Table 1).

### Geographic distribution

Distribution of retinoblastoma by regions showed 38.9% of patients residing in Oromia, the most populous region in the country, followed by Amhara at 19.9%. Addis Ababa city and Southern Nations, Nationalist and People Region (SNNPR) followed with 16.7% and 14.4%, respectively. There were no patients originating from Dire Dawa city (shown in Table 2).

The sex distribution per region was variable, with the highest male to female ratio observed in the SNNPR (shown in Table 2). The highest number of unilateral RB cases were reported from Amhara region. Zonal (Provincial) level distribution of retinoblastoma cases are shown in Supplementary File 1.

**Table 1:** Study population Demographical Data Distribution, N = 221

		All Years	2017	2018	2019	2020
		221 (100%)	91 (41.2%)	53(24%)	62(28.1%)	15(6.8%)
**Sex**	Male (n, %)	115 (52%)	54(59.3%)	27 (23.5%)	31 (26.9%)	3 (2.6%)
	Female	106 (48%)	37(34.9%)	26 (24.5%)	31(29.2%)	12 (11.3%)
**Laterality**	Unilateral	185 (83.7%)	80 (43.2%)	42(22.7%)	49 (26.5)	14 (7.6%)
	Bilateral	36 (16.3%)	11 (30.6%)	11(30.6%)	13 (36.1%)	1 (2.8%)
**Family History**	Yes	11 (5%)	6 (54.5%)	3 (27.3%)	2 (18.2%)	0 (0%)
	No	210 (95%)	85 (40.5%)	50 (23.8%)	60 (28.6%)	15 (7.1%)
**RB Centers**	Menelik II	159 (71.9%)	72 (45.3%)	35 (22%)	47 (29.6%)	5 (3.1%)
	Jimma University Hospital	32 (14.5%)	9 (28.1%)	7 (21.9%)	11 (34.4%)	5 (15.6%)
	University of Gondar Hospital	20 (9%)	8 (40%0	6 (30%)	3 (15%)	3 (15%)
	Hawassa University Hospital	10 (4.5%)	2 (20%)	5 (50%)	1 (10%)	2 (20%)
**Region of Origin**	Tigray	6 (2.7%)	5 (83.3%)	1 (16.7%)	0 (0%)	0 (0%)
	Afar	1 (0.5%)	1 (100%)	0 (0%)	0 (0%)	0 (0%)
	Amhara	44 (19.9%)	18 (40.9%)	11 (25%)	11 (25%)	4 (9.1%)
	Oromia	86 (38.9%)	40(46.5%)	17 (19.8%)	25 (29%)	4 (4.7%)
	Somali	3 (1.4%)	1 (33%)	1 (33%)	1 (33%)	0 (0%)
	Benishangul Gumuz	2 (0.9%)	1 (50%)	1 (50%)	0(0%)	0(0%)
	SNNPR	32 (14.5%)	7 (21.9%)	11 (34.4%)	10 (31.3%)	4 (12.5%)
	Gambella	7 (3.2%)	4 (57.1%)	1 (14.3%)	2 (28.6%)	0 (0%)
	Harari	3 (1.4%)	2 (66.7%)	0 (0%0	1(33.3%0	0 (0%0
	Addis Ababa	37 (16.7%)	12 (32.4%)	10 (27%)	12 (32.4%)	3 (8.1%)

**Table 2:** Distribution of Retinoblastoma cases by regional states in Ethiopia ,2017–2020 (n = 221)

Region	Retinoblastoma Cases		Sex		Laterality	
	n	%	M	F	Unilateral	Bilateral
Tigray	6	2.7%	2	4	5	1
Afar	1	0.5%	1	0	1	0
Amhara	44	19.9%	21	23	33	11
Oromia	86	38.9%	45	41	77	9
Somali	3	1.4%	2	1	3	0
Benishangul Gumuz	2	0.9%	1	1	2	0
SNNPR	32	14.5%	22	10	24	8
Gambella	7	3.2%	3	4	6	1
Harari	3	1.4%	1	2	2	1
Addis Ababa city	37	16.7%	17	20	32	5
Total	221	100.0%	115	106	185	36

### Incidence by Birth Cohort Analysis

Incidence was calculated for study subjects within the 2017-2019-time period, as population data was not available for 2020. Accordingly, incidence of RB in 2017 was 1:36,807 live births; in 2018, 1:64,112 live births, and in 2019, 1 in 55,548 live births. Average incidence for the 3 years was 1 in 52,156 live births (shown in Table 3).

**Table 3:** Incidence of retinoblastoma in Ethiopia by year

Year	Population	Birth rate	Infant Mortality rate	Live birth	No of RB cases born in	Incidence	3 year average incidence
2017	106,399,926	32.775	39.5	3,349,511	91	1:36807	1:52156
2018	109,224,410	32.339	38	3,397,984	53	1:64112	
2019	112,078,727	31.896	36.6	3,444,023	62	1:55548	

## Discussion

This study evaluated the incidence and geographical distribution of patients with RB from 2017 to 2020 at the four main RB treatment centers in Ethiopia. This study on incidence and distribution of RB is the first of its kind in Ethiopia and one of the few studies from Sub-Saharan Africa.

The equal distribution of retinoblastoma with regard to sex is consistent with prior literature with respect to the biological development of disease [14–18]; in some cultures, sex of the child has been shown to influence parental health-seeking behaviour, but that did not appear to be a factor in our study. That 83.7% of patients presented with unilateral disease is inconsistent with global figures suggesting it is closer to 60% [19], but consistent with a previous study from Ethiopia [20]. About 5% of the total study population had a positive family history, which is consistent with published literature [19, 21].

Our study included centers in different geographic locations and catering all referrals from all over the country. These four centers are believed to cater to all RB referrals, hence we assumed that all RB cases nationwide would present to one of the study sites and be captured in the study. However, our findings suggest that we missed a large proportion of expected RB patients. Based on an estimated uniform annual RB incidence of 1 in 16,000–18,000 live births using birth cohort analysis [8, 9], Ethiopia might expect observe 193–217 new RB patients per year [13]. Yet, the cases seen by the participating 4 centres for the period of 2017–2020 yielded an incidence of 1 in 52,156 live births. The cases reported in our study about 33.9–38.2% of the predicted cases of RB for the study period. One reason for this underrepresentation is that RB patients with advanced stage at presentation are often directly referred to a center that can offer chemotherapy; in many instances, pediatric ophthalmology and pediatric oncology units are in separate hospitals. As our study collected records from pediatric ophthalmology units only, it is possible that some cases were either not seen in those units, or not adequately documented prior to referral to a pediatric oncology unit.

We also observed a difference in the incidence of retinoblastoma among the regional states s indicating a lower than expected incidence for children from Tigray, Afar, Somali and Benishangul Gumuz states. These regions account for 14.7% of total at risk population in Ethiopia, but contributed only 5% of the total RB cases reported in this study. This could partially be explained by the long distance required for patients from these regions to access health services. Distance from hospital has been shown to contribute to diagnostic and treatment delays for many Ethiopian patients [22], thus it not a far reach to suggest distance may preclude presentation at an RB treatment facility.

Hospital based registries tend to underestimate the incidence of RB, Studies in India [23, 24], Taiwan [25], China [26, 27], Indonesia [28], Iran [29, 30], Pakistan [31], and the Philippines [32] documented 5–35% of their total expected cases. This limitation can be surpassed in retrospective studies by ensuring comprehensive data collection from all facilities where RB patients may present. One such study from Kenya, which collected data from primary, secondary and tertiary level facilities, managed to capture all expected RB cases in the country [21]. Better still, systematic collection of prospective retinoblastoma data through nationwide cancer registries shows the most potential to facilitate comprehensive data capture. Work is already underway to test the roll-out of a national pediatric cancer registry in Ethiopia [33]. Still, it will be important to coordinate efforts between pediatric ophthalmology and oncology units to ensure all RB patients are captured.

Though we were unable to calculate incidence for the year 2020, we noticed a sharp decline in the number of RB patients seen in 2020 as compared to the previous years (shown in Table 1). This could partially be explained by the COVID-19 pandemic, which affected both the healthcare delivery system and health seeking behaviour. In Ethiopia a cross-sectional hospital-based telephone survey during the COVID-19 pandemic indicated that a loss to follow-up, missed medication and death occurred in 70%, 12%, and 1.3% of patients with chronic medical conditions respectively [34]. A global study of RB trends during the pandemic indicated that families faced restrictions in traveling to medical centers for care [35]. It is also possible that the political instability in the country, which began in 2018, contributed to the observed decline.

### Limitations

The unavailability of recent population and housing census data was the main limitation of this study. We initially planned to obtained this data from Ethiopian Statistical Agency, but used an alternate from the World Bank data to compensate for the missing local data. In addition, the collected data is retrospective in nature from hospital-based registry was used and some vital data required for analysis were missing which includes religion and staging of the disease.

We included centers in different geographic locations and catering all referrals from all over the country. The four centers located at different directions in the country are believed to cater all RB referrals and we believe all Retinoblastomas in the study period presented to one of the centers and captured in the study.

## Conclusions

The national retinoblastoma incidence calculated by our paper is lower than global estimates. More accurate assessment of RB incidence would be facilitated through the implementation a nationwide cancer registry. Our work also suggests that patients may not be reaching treatment facilities, revealing an urgent need for capacity improvements that will avail RB treatment to patients residing in underserved areas of Ethiopia.

## Electronic supplementary material

Below is the link to the electronic supplementary material.

Supplementary Material 1

## Data Availability

All data generated or analysed during this study are included in this published article and its supplementary information files.

## Data Availability

All data generated or analysed during this study are included in this published article and its supplementary information files.
