# Supplementary material for: Incidence and geographic distribution of retinoblastoma in Ethiopia
Source: BMC Ophthalmol. 2023 May 23;23:231. doi: 10.1186/s12886-023-02980-8 (PMC10203669; doi:10.1186/s12886-023-02980-8)
Supplement: Supplementary file 1 — Supplementary Material 1 [file 12886_2023_2980_MOESM1_ESM.doc]

## Supplementary file. Distribution of Retinoblastoma by Zones (Provinces) (n=221)

| **Region** | **Zone** | **No of RB** | **Total** |
| --- | --- | --- | --- |
| Tigray | Debubawi zone | 1 | 6 |
|  | Mekele special zone | 2 |  |
|  | Mierabawi zone | 1 |  |
|  | Misrakawi zone | 2 |  |
| Afar | Gabi Rasu | 1 | 1 |
| Amhara | North Gondar | 4 | 44 |
|  | South Gondar | 6 |  |
|  | North Wollo | 4 |  |
|  | South Wollo | 1 |  |
|  | North Shewa | 4 |  |
|  | East Gojjam | 9 |  |
|  | West Gojjam | 9 |  |
|  | Agew/Awi | 3 |  |
|  | Oromia special | 2 |  |
|  | Bahirdar special zone | 1 |  |
|  | Argobba | 1 |  |
| Oromia | Arsi | 15 | 86 |
|  | Bale | 6 |  |
|  | Buno Bedelle | 3 |  |
|  | East Harerge | 2 |  |
|  | East Shewa | 5 |  |
|  | East Wollega | 4 |  |
|  | Guji | 2 |  |
|  | Horo Guduru Wollega | 5 |  |
|  | Iluababor | 2 |  |
|  | Jimma | 21 |  |
|  | Kelem Wollega | 1 |  |
|  | North Shewa | 4 |  |
|  | Southwest Shewa | 6 |  |
|  | West Arsi | 2 |  |
|  | West Harerge | 1 |  |
|  | West Shewa | 4 |  |
|  | West Wollega | 1 |  |
|  | Finfinnee Special Zone | 2 |  |
| Somali | Fafan | 3 | 3 |
| SNNPR | Dawro | 2 | 32 |
|  | Gamo | 6 |  |
|  | Gofa | 2 |  |
|  | Gedeo | 3 |  |
|  | Gurage | 5 |  |
|  | Hadiya | 4 |  |
|  | Keffa | 4 |  |
|  | Sheka | 1 |  |
|  | South Omo | 1 |  |
|  | Wolayta | 1 |  |
|  | Alaba | 3 |  |
| Gambella | Anywaa | 7 | 7 |
| Benishangul Gumuz | Assosa | 2 | 2 |
| Harari | Harari | 3 | 3 |
| Addis Ababa City | Addis Ababa | 37 | 37 |
| Total |  | 221 | 221 |
